# Down Regulation of the Expression of ELMO3 by COX2 Inhibitor Suppresses Tumor Growth and Metastasis in Non-Small-Cell Lung Cancer

**DOI:** 10.3389/fonc.2019.00363

**Published:** 2019-05-07

**Authors:** Cailong Pan, Yong Zhang, Qinghai Meng, Guoliang Dai, Zhitao Jiang, Hongguang Bao

**Affiliations:** ^1^Department of Anesthesiology, Nanjing First Hospital, Nanjing Medical University, Nanjing, China; ^2^School of Pharmacy, Nanjing University of Chinese Medicine, Nanjing, China; ^3^Department of Clinical Pharmacology, Affiliated Hospital of Nanjing University of Chinese Medicine, Nanjing, China; ^4^Department of Pharmacy Office, Zhangjiagang Hospital of Traditional Chinese Medicine Affiliated to Nanjing University of Chinese Medicine, Zhangjiagang, China

**Keywords:** ELMO3, COX-2, EMT, NSCLC, ParecoxibNa

## Abstract

Non-small cell lung cancer (NSCLC) is one of the most common malignancies. Studies have shown that engulfment and cell motility 3 (ELMO3) is highly expressed in NSCLC and can be used as a novel biomarker, but its underlying mechanism remains to be explored. The aim of this study was to investigate the mechanism by which ELMO3 may be down-regulated by COX-2 inhibitors to inhibit NSCLC. NSCLC tissue and adjacent normal lung tissue from 24 patients were used to detect the mRNA and protein expression of ELMO3, COX-2, and other related proteins by Western blot, RT-PCR, and Immunohistochemical analysis. Lewis Lung carcinoma (LLC) cells were used to investigate the effects and the mechanism of siELMO3 and COX-2 inhibitor. C57BL/6 mice inoculated with LLC cells by subcutaneous (s.c.) injection were used to detect the *in vivo* effects of cox-2 inhibitor. The expression of ELMO3 and cyclooxygenase-2 (COX-2) in human NSCLC tissues was significantly increased compared with that in the adjacent normal tissues. ELMO3 exhibited a positive correlation with COX-2 expression. Moreover, knockdown of ELMO3 suppressed the epithelial-mesenchymal transition (EMT), adhesion, and metastasis of Lewis lung carcinoma (LLC) cells. Importantly, Parecoxib, a selective inhibitor of COX-2, significantly reduced the expression of ELMO3 and EMT in LLC cells and LLC-bearing mice. Furthermore, it could inhibit the growth, adhesion and metastasis of LLC cells *in vitro*. Our results demonstrate that down regulation of ELMO3 suppressed growth and metastasis of lung cancer by inhibiting EMT. Parecoxib could reduce ELMO3 expression and suppress growth and metastasis of lung cancer, which might be a useful chemotherapeutic agent for inhibiting metastasis and recurrence of NSCLC.

## Introduction

Lung cancer is one of the most serious malignancies that threaten people's health and life. Eighty percent of lung cancer belongs to non-small cell lung cancer (NSCLC), which is the main cause of lung cancer-related death (1). Surgical resection is a common therapeutic strategy at the early stages, however, more than 50% of patients have metastasis within 1 year after surgery (2, 3). Therefore, new targets and therapeutic drugs are needed for the treatment of NSCLC.

Studies found that engulfment and cell motility (ELMO) plays an important role in the remodeling of the cytoskeleton and promoting the movement and invasion of tumor cells (4–6). This family of proteins is evolutionarily highly conserved and includes ELMO1, ELMO2, and ELMO3, major structures of these three are highly similar. Among them, ELMO1 and ELMO2 are widely expressed in a variety of tumors, including glioma, ovarian cancer, and liver cancer, involvement in tumor development, invasion, metastasis, and prognosis (7–9). ELMO3, however, is only reported to be highly expressed in early glottic cancer, lung cancer and colorectal cancer (10–12). The study found that ELMO3 knockdown inhibited colorectal cancer growth and metastasis (12). The results of studies in lung cancer suggest that ELMO3 protein is a potential diagnostic and prognostic marker for NSCLC (13). These studies suggest that down regulation of the expression of ELMO3 may be an effective treatment for NSCLC, and safe and effective ELMO3 inhibitors that can be used clinically are urgently needed.

Parecoxib is a cyclooxygenase-2 (COX-2) specific inhibitor for the treatment of moderate or severe peri-operative acute pain. Evidence shows that COX-2 can be used as an index to judge the prognosis of lung cancer. Knockout of COX-2 gene can significantly inhibit NSCLC growth and induce tumor cell growth cycle arrest (14). However, whether COX-2 inhibitors can inhibit NSCLC by downregulating ELMO3 has not been reported.

Here, we compared the different expression of ELMO3 with normal and NSCLC tissue in human and mouse samples and investigate the association with ELMO3 and COX2. Furthermore, we also discussed that whether ELMO3 might be modulated by Parecoxib, a COX2 inhibitor, for reducing tumor cell metastasis and recurrence of NSCLC.

## Materials and Methods

### Tissue Samples

All 24 patients who provided NSCLC and adjacent normal lung tissue lobectomy specimens provided full consent for the study. All patients had not received antitumor drugs or radiotherapy before surgery. This study followed the tenets of the Declaration of Helsinki, and informed written consent was obtained from all patients after clinicians explained the purpose, nature and possible consequences of the study.

### Reagents

ParecoxibNa was purchased from Pfizer (Pfizer Inc., New York, USA). Antibodies for ELMO3, COX-2, E-cadherin, Snail, N-cadherin, and Vimentin were purchased from Abcam (CA, USA). Antibody for glyceraldehyde-3-phosphate dehydrogenase (GAPDH) was from Sigma-Aldrich (St. Louis, MO, USA). CCK8 kit was purchased from Biosharp (Hefei, China).

### Hematoxylin-Eosin (H & E) Staining

Briefly, paraffin embedded tissue specimens were cut into 4 μm slices, dewaxed and hydrated. It was stained with hematoxylin for 4 min and eosin for 90 s. It was dehydrated with an alcohol gradient from low to high concentrations, transparent with xylene, and then observed under the microscope.

### Western Blot

Tissues or cells were placed in a pre-cooled glass grinder, and a pre-cooled lysate containing PMSF and phosphatase inhibitors was added and thoroughly ground and lysed. Appropriate amount of 5X protein loading buffer was added to the protein sample. The protein was separated by SDS-PAGE and transferred to PVDF membranes. The membranes were blocked with 5% bovine serum albumin at room temperature for 1 h and then incubated with a specific primary antibody overnight at 4°C. The primary antibodies used included glyceraldehyde 3-phosphate dehydrogenase, 1:5,000; ELMO3, 1:500; COX-2, 1:500; E-cadherin 1:1,000; Snail 1:500, N-cadherin 1:1,000, and Vimentin 1:1,000. The corresponding secondary antibody was incubated for 1 h at room temperature and then the blot was visualized with enhanced chemiluminescence (ECL) method. Data were analyzed with the Molecular Imager (Gel DocTM XR, 170–8170) and the associated software Quantity One-4.6.5 (Bio-Rad Laboratories, USA).

### Cell Culture

Lewis Lung carcinoma (LLC) cells were cultured in DMEM containing 10% fetal bovine serum (FBS), 100 U/mL penicillin, and 100 mg/mL streptomycin and incubated at 37°C in an incubator containing 5% CO_2_. ParecoxibNa was dissolved in saline and cells were treated with different concentrations of ParecoxibNa (250, 500, and 1,000 μg/ml) for 24 h.

### CCK Assay

The CCK-8 kit was used to detect cell proliferation. Briefly, LLC cells were seeded in 96-well plates at 2 × 10^4^ cells/well and after 24 h the medium was changed to medium containing different concentrations of ParecoxibNa (250, 500, and 1,000 μg/ml) for 24 h. The medium was discarded and PBS was added, followed by incubation with 10 μl CCK-8 for 0.5 h. Absorbance was measured at 450 nm using a microplate reader (Biorad, USA).

### Cell Adhesion Assays

In brief, LLC cells were treated with different concentrations of ParecoxibNa (250, 500, 1,000 μg/ml) for 24 h. Before adhesion, 96-well plates were treated with FN (20 μg/ml) -coated for 30 min. Then Cells (1 × 10^4^ cells/well) were added to FN-coated 96-well plates and labeled with FDA (5 μg/ml) for 20 min; unbound cells were removed by 2 washes with PBS. Immunofluorescence images were taken with a DMI3000B inverted microscope (Leica, Germany).

### Transwell Assays

In brief, serum-free medium containing different concentrations of ParecoxibNa (250, 500, and 1,000 μg/ml) was added to the upper chamber of the transwell (Corning, USA), and the complete medium was added to the lower chamber and cultured for 24 h. The cells in the upper chamber were removed and the cells on the underside of the membrane were fixed, stained with crystal violet and observed under the microscope.

### Real Time-PCR

Trizol reagent was used to extract total RNA according to the manufacturer's instructions and cDNA was synthesized. PCR experiments were then performed with ABI7500 (Life Technologies). The primers used for the analysis of ELMO3, COX-2, and GAPDH expression were as follows. ELMO3 (human): 5′- CGGACATGATCTTTGCCAGG-3′, and 5′- CCTAGGTCGTCCCCATCTTC-3′. COX-2 (human): 5′- GAATGGGGTGATGAGCAGTT-3′ and 5′- CAGAAGGGCAGGATACAGC-3′. GAPDH (human): 5′- ACCCACTCCTCCACCTTTGA-3′ and 5′- CTGTTGCTGTAGCCAAATTCGT-3′. ELMO3 (mouse): 5′- TAATACGACTCACTA TAG GG-3′ and 5′- TAGAAGGCACAGTCGAGG-3′. GAPDH (mouse): 5′- CCCATCACCATCTTCCAGGAGC-3′, and 5′- CCAGTGAGCTTCCCGTTCAGC -3′.

### ELMO3 siRNA Transfection

LLC cells were transfected with ELMO3 siRNA and negative control (NC) siRNA using LipofectamineTM 2000 reagent for 24 h.

### Mouse Tumor Models

C57BL/6 mice were anesthetized and 3 x 10^5^ LLC cells were injected subcutaneously (s.c.) on the right flank. Four days after LLC vaccination, each mouse was injected with ParecoxibNa (2.5, 5, and 10 mg/kg, i.p.) daily for 7 d. Control mice were administered saline. The tumor volume was measured with a caliper every 2 d and the tumor volume was calculated according to the formula [(length × width^2^)/2]. Twenty one days after inoculation of LLC cells, all mice were sacrificed and the tumors were removed and weighed.

### Statistical Analysis

Statistical analysis was carried out using *t*-test or ANOVA. Results are expressed as mean ± SD. *p* < 0.05 was considered as a significant difference. All analyses were performed with GraphPad Prism Version 5.01 (GraphPad Software Inc., San Diego, CA, USA).

## Results

### Positive Correlation Between ELMO3 and COX-2 Expression in NSCLC Tissues

**Figure 1:**
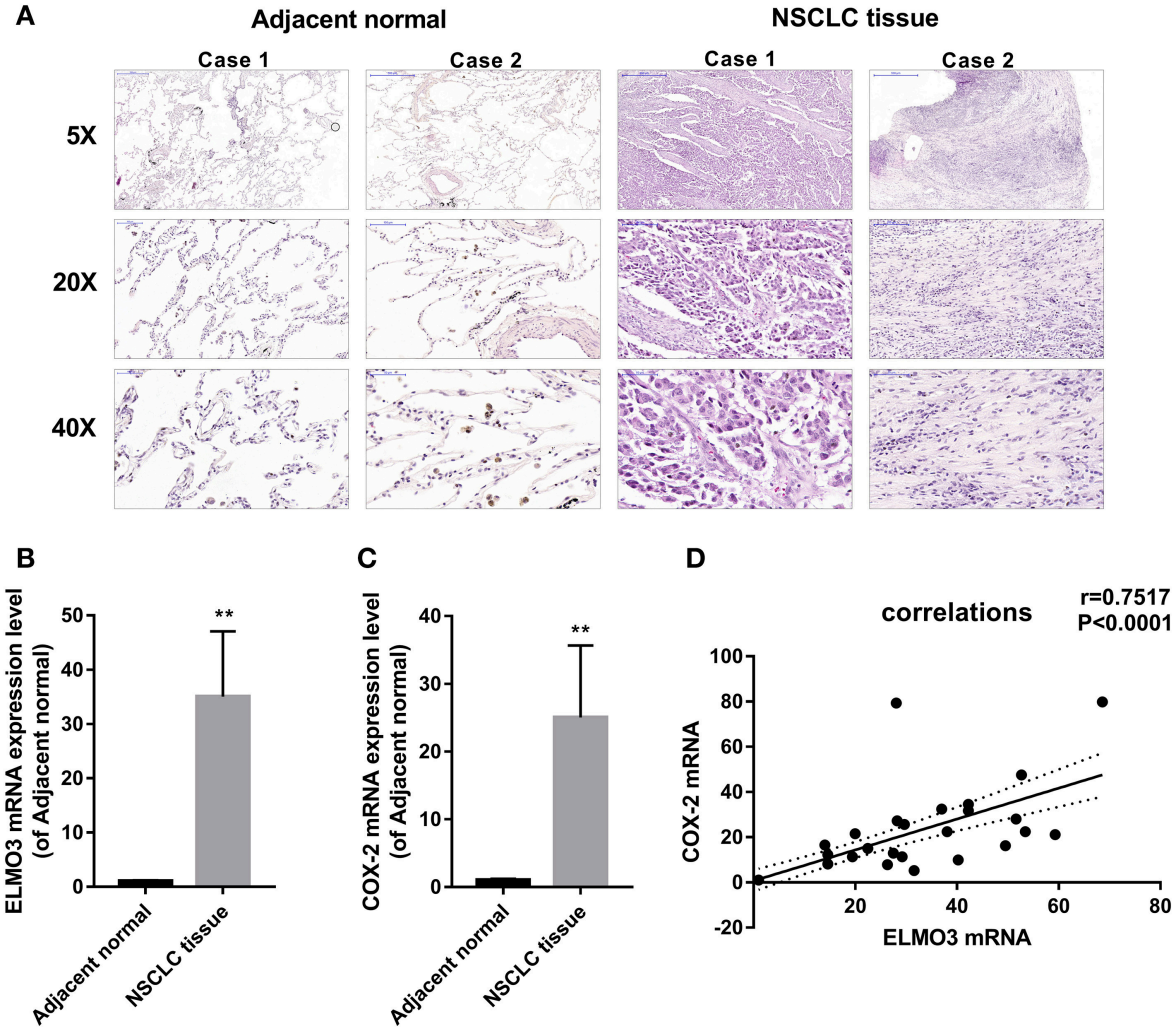
Positive correlation between ELMO3 and COX2 in human non-small-lung cancer tissue. **(A)** Hematoxylin and eosin (H&E) staining of NSCLC tissues and human adjacent normal tissues from patients with NSCLC. Original magnification: 5, 20, or 40. **(B)** RT-PCR analysis of ELMO3 mRNA levels in NSCLC tissues and human adjacent normal tissues. **(C)** RT-PCR analysis of COX-2 mRNA levels in NSCLC tissues and human adjacent normal tissues. **(D)** Correlation of ELMO3 and COX-2 expression was examined by RT-PCR in 24 cases of clinical NSCLC tissues (Pearson's correlation coefficient, *r* = 0.7517). **P < 0.01, vs. Adjacent normal.

First, we analyzed human NSCLC tissues and adjacent normal lung tissues. The H&E analysis of 24 pairs of human samples demonstrated moderately differentiated adenocarcinoma (Figure 1A). RT-PCR analysis revealed that ELMO3 was significantly increased in human NSCLC tissues, compared with adjacent normal lung samples, as was COX-2 (Figures 1B,C). Moreover, the correlation analysis determined that ELMO3 expression was positively correlated with COX-2 expression in these tissues (Figure 1D), suggesting that ELMO3 might be involved in COX-2 downstream signaling in NSCLC.

### Knockdown of ELMO3 Suppressed the Growth, Adhesion, Metastasis, and EMT of LLC Cells

**Figure 2:**
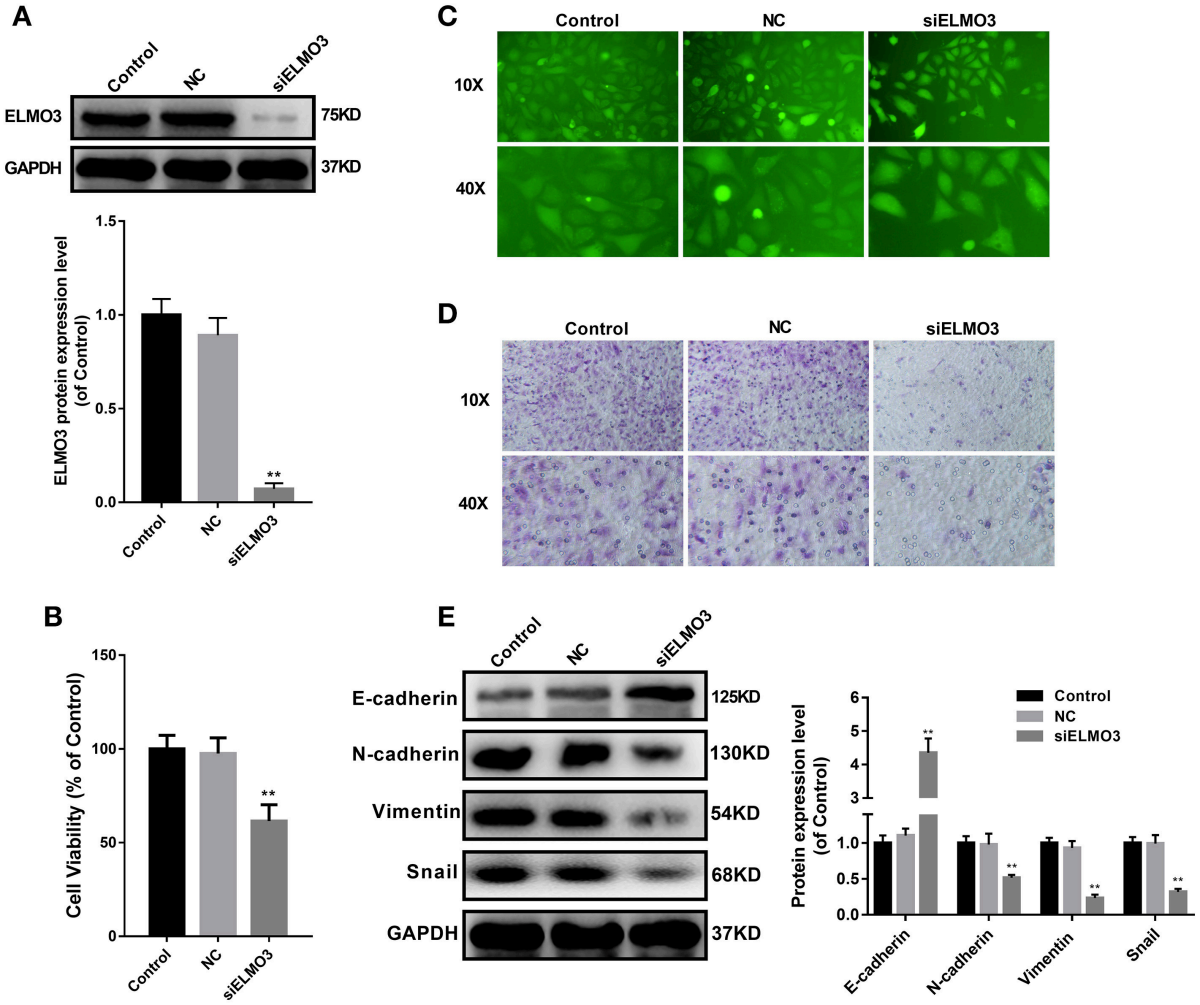
siELMO3 suppressed cell viability, adhesion, metastasis and EMT of LLC cells. **(A)** The inhibition efficiency of the ELMO3 siRNA was determined by Western blot analysis. **(B)** Cytotoxicity of Parecoxib in LLC cells. Cell viability was determined by CCK8 assay after a 24 h treatment. **P < 0.01 vs. the NC group. **(C)** LLC cells treated with siELMO3 or NC siRNA were applied for the adhesion experiment. **(D)** LLC cells treated with siELMO3 or NC siRNA were applied for the trasnwell experiment. **(E)** E-cadherin, N-cadherin, Snail and Vimentin were detected by Western blot in LLC cells after treatment with siELMO3. GAPDH was used as an internal control. **P < 0.01 vs. the NC group.

EMT plays an important role in the migration, invasion and metastasis of many types of cancer, including NSCLC. During EMT, the expression of E-cadherin protein is decreased, leading to a lost connection between epithelial cells. Increased expression of N-cadherin, Vimentin, and Snail increases migratory and invasive properties. To test whether knockdown of ELMO3 could suppress EMT, we tested the expression of E-cadherin, N-cadherin, Snail, and Vimentin by western blot. First, we silenced ELMO3 with ELMO3 siRNA transfection (Figure 2A). Data presented in Figure 2E indicated that the silencing of ELMO3 could significantly up-regulate the expression of the E-cadherin and down-regulate the expression of N-cadherin, Snail, and Vimentin, compared with negative control (NC) siRNA. Moreover, siELMO3 could significantly suppress the viability of LLC cells compared with the NC group by CCK8 assays (Figure 2B). In addition, the adhesion experiment showed that siELMO3 significantly inhibit the adhesion ability of LLC cells (Figure 2C), consistent with the control group, and the transwell assay also revealed a similar result (Figure 2D).

### Parecoxib Reduced ELMO3 Expression in LLC Cells

**Figure 3:**
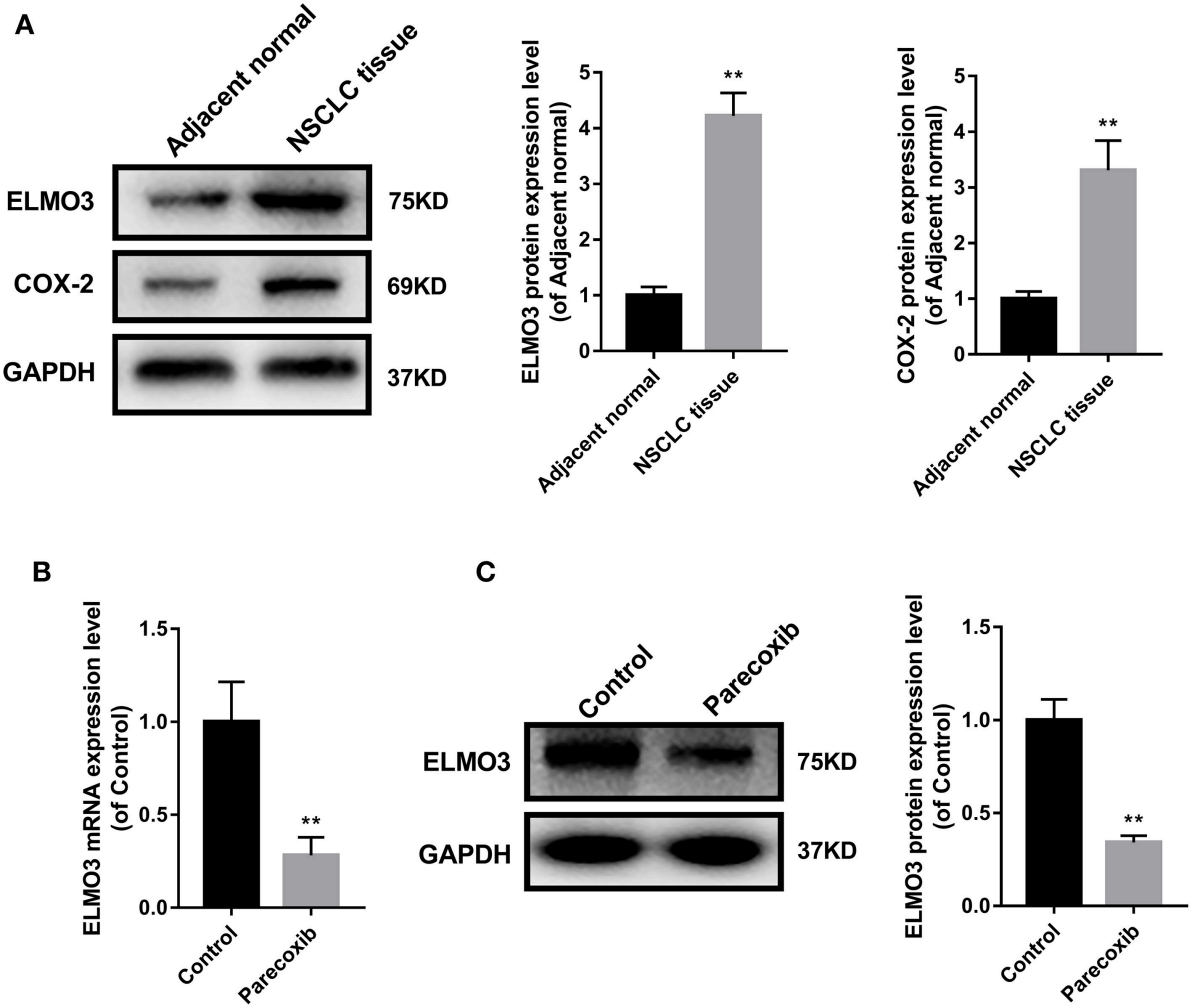
Parecoxib inhibit ELMO3 expression in LLC cells. **(A)** ELMO3 and COX2 expression in human tissue samples. The protein levels of ELMO3 and COX2 were detected by western blot. *P < 0.05, **P < 0.01, vs. Adjacent normal. **(B)** The effects of Parecoxib on mRNA expression levels of ELMO3 in LLC cells. GAPDH was used as a loading control. The mRNA level of ELMO3 was detected by RT-PCR. **P < 0.01 vs. the control group. **(C)** The effects of Parecoxib on the expression of ELMO3 in LLC cells. The protein level of ELMO3 was detected by western blot. **P < 0.01 vs. the control group.

The above studies confirmed that ELMO3 was positively correlated with COX-2 and siELMO3 could inhibit EMT. We next investigated whether COX-2 inhibitor could reduce the expression of ELMO3. Western blot analysis revealed that ELMO3 was significantly increased in human NSCLC tissues, compared with adjacent normal lung samples, as was COX-2 (Figure 3A). The data presented in Figure 3B demonstrate that COX-2 inhibitor Parecoxib markedly reduced the mRNA expression of ELMO3 in LLC cells. Moreover, Parecoxib significantly suppressed the protein expression of ELMO3 in LLC cells (Figure 3C).

### Parecoxib Inhibited EMT of LLC Cells

**Figure 4:**
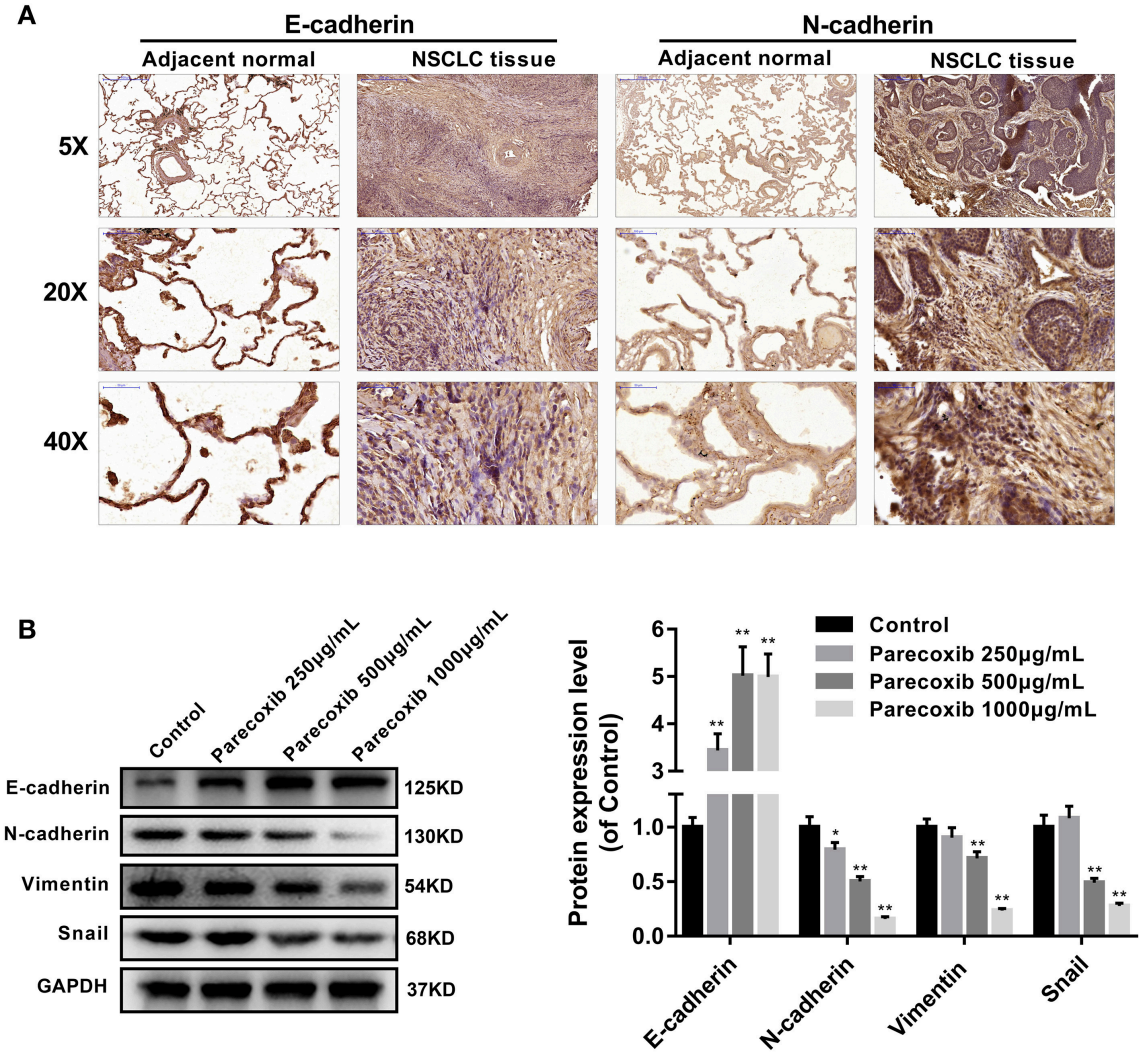
Parecoxib inhibited EMT of Lewis cells. **(A)** Immunohistochemical staining of E-cadherin and N-cadherin in human tissue samples. **(B)** E-cadherin, N-cadherin, Snail, and Vimentin were detected by Western blot in LLC cells after treatment with Parecoxib (0, 250, 500, and 1,000 μg/ml) for 24 h. GAPDH was used as an internal control. *P < 0.05, **P < 0.01 vs. the control group.

Immunohistochemistry analysis revealed that E-cadherin was significantly decreased, while N-cadherin was increased in human NSCLC tissues, compared with adjacent normal lung samples (Figure 4A). We next examined the ability of COX-2 inhibitor Parecoxib to inhibit EMT in LLC cells. LLC cells were treated with different concentration of Parecoxib (0, 250, 500, and 1,000 μg/mL) for 24 h. The results indicated Parecoxib could significantly upregulate the expression of the E-cadherin and downregulate the expression of N-cadherin, Snail, and Vimentin by Western blot (Figure 4B). These data suggest that Parecoxib effectively prevent EMT in a dose-dependent manner in LLC cells.

### Parecoxib Inhibited the Growth, Adhesion, and Metastasis of LLC Cells

**Figure 5:**
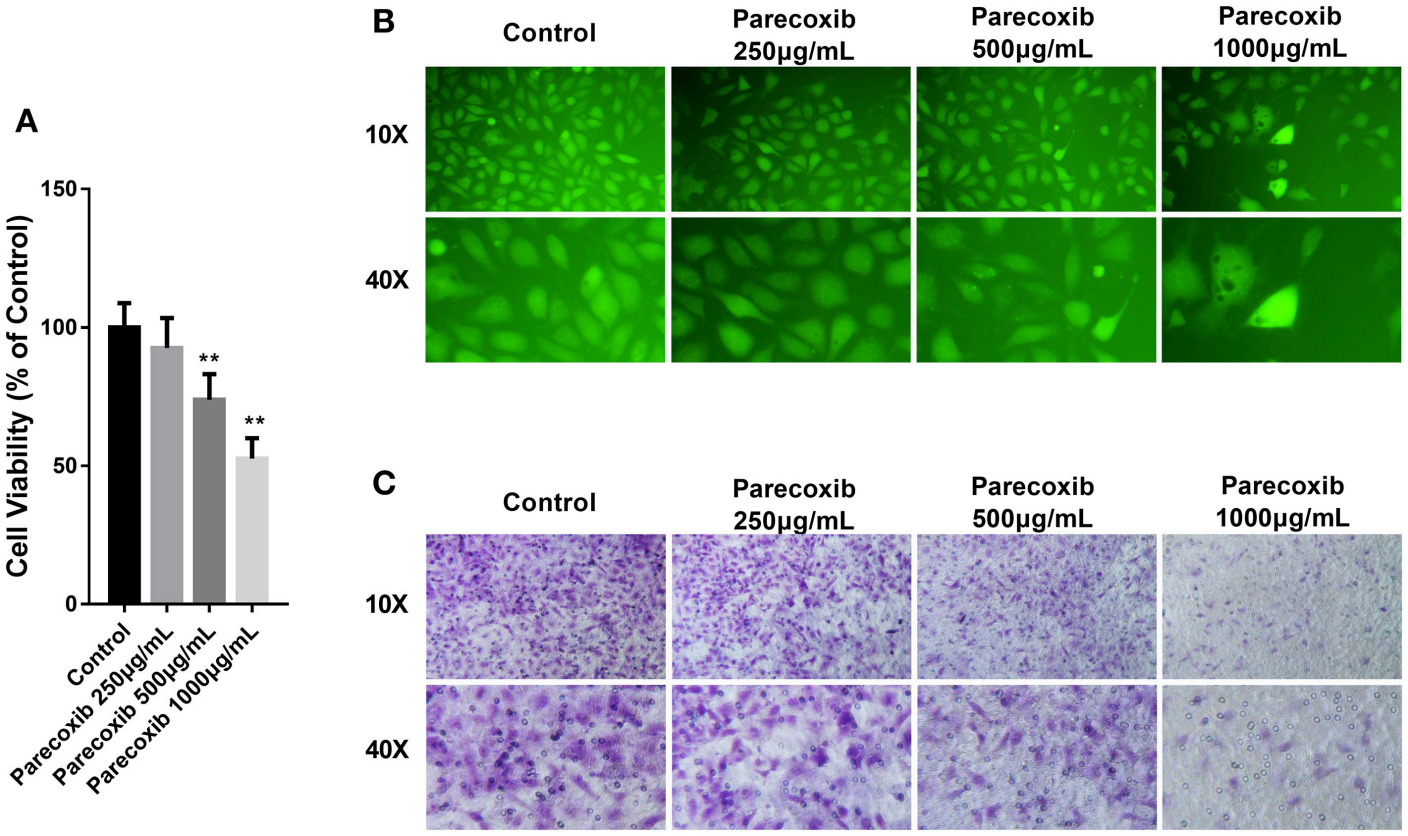
Parecoxib inhibited cell viability, adhesion, and metastasis in LLC cells. **(A)** Cytotoxicity of Parecoxib in Lewis cells. Cell viability was determined by CCK8 assay after a 24 h treatment. **P < 0.01 vs. the control group. **(B)** LLC cells treated with or without Parecoxib (0, 250, 500, and 1,000 μg/ml) were applied for the adhesion experiment. **(C)** LLC cells treated with or without Parecoxib (0, 250, 500, and 1,000 μg/ml) were applied for the trasnwell experiment.

Then we investigated whether COX-2 inhibition could mimic the effect of siELMO3 in LLC cells. We examined the effect of Parecoxib on LLC cells. Parecoxib could significantly dose- dependently suppress LLC cell viability compared with the control group by CCK8 assays (Figure 5A). Also, the adhesion experiment showed that Parecoxib inhibited the adhesion ability of LLC cells, consistent with the control group (Figure 5B). Moreover, by the transwell assay, we found that Parecoxib could inhibit the migratory ability of LLC cells significantly (Figure 5C). These results indicate that Parecoxib could inhibit the growth, adhesion and metastasis of LLC cells *in vitro*.

### Parecoxib Inhibited the Expression of ELMO3 and EMT in LLC-Bearing Mice

**Figure 6:**
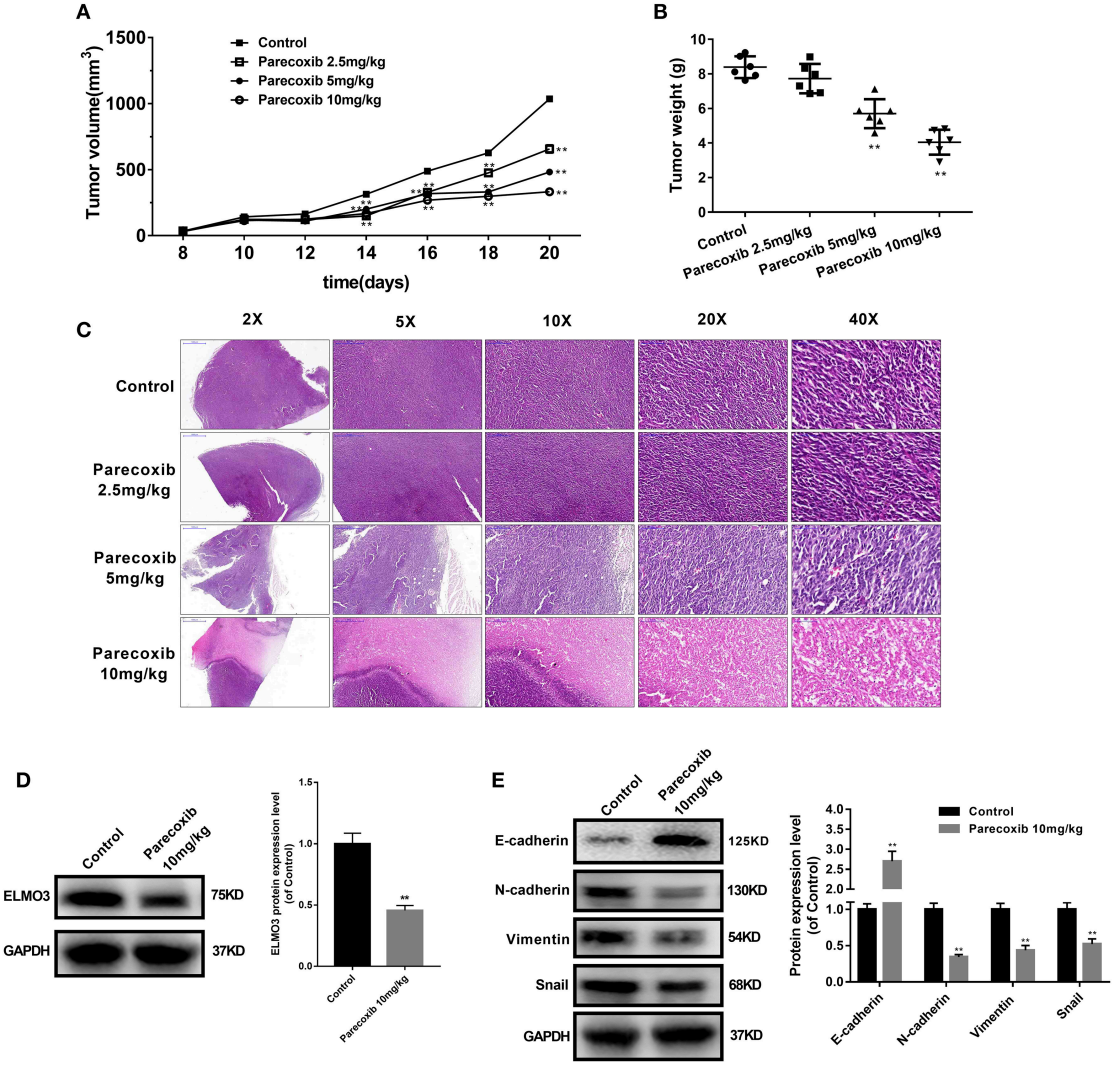
Parecoxib down-regulated the expression of ELMO3 and inhibited the tumor growth and metastasis *in vivo*. LLC cells were injected subcutaneously in the right flanks of C57BL/6 mice. LLC-bearing mice (*n* = 6 per group) were injected with PBS (control) and Parecoxib (2.5, 5, and 10 mg/kg body weight) for 21 days. **(A)** Tumor size was measured every 2 days after tumor cell inoculation for 8 days in each group. **(B)** Tumor weight was measured after treatment for 21 days in each group. **P < 0.01 vs. the control group. **(C)** Pictures of mouse tumor segments stained with H&E in the control and Parecoxib treatment groups. HE staining of tumor segments. **(D)** The effects of Parecoxib on the expression of ELMO3 in LLC-bearing mice. The protein level of ELMO3 was detected by western blot. **P < 0.01 vs. the control group. **(E)** E-cadherin, N-cadherin, Snail and Vimentin were detected by Western blot after treatment with Parecoxib. GAPDH was used as an internal control. **P < 0.01 vs. the control group.

To further investigate the inhibition of Parecoxib in ELMO3 *in vivo*, LLC cells were inoculated into C57BL/6 mice. The tumor volumes of Parecoxib treatment groups were markedly smaller than those of control group (Figure 6A). Parecoxib significantly reduced tumor weight in a dose-dependent manner in LLC-bearing mice (Figure 6B). The H & E analysis of Parecoxib treatment groups and model group of tumor samples demonstrated that tumor growth was inhibited (Figure 6C). Then we measured the expression of ELMO3. Western blot analysis revealed that Parecoxib significantly inhibited the expression of ELMO3, which was consistent with the *in vitro* data (Figure 6D). Moreover, Parecoxib could significantly upregulate the expression of the E-cadherin and downregulate the expression of N-cadherin, Snail and Vimentin by Western blot (Figure 6E), suggesting that Parecoxib effectively prevented EMT in LLC-bearing mice. These data showed that Parecoxib significantly inhibited the tumor growth and metastasis via downregulation of ELMO3.

## Discussion

Our study demonstrated that ELMO3 exhibited a positive correlation with COX-2, and both were increased in human NSCLC tissues. Knockdown of ELMO3 suppressed the EMT, adhesion, and metastasis of LLC cells. Parecoxib could inhibit the expression of ELMO3 *in vitro* and *in vivo*. Moreover, Parecoxib could significantly inhibit the growth, adhesion and metastasis of LLC cells. Taken together, these results may enhance our understanding of Parecoxib against NSCLC through ELMO3 and provide alternative option for the future treatment of NSCLC.

The ELMO family plays an important role in the regulation of actin-regulating processes such as chemotaxis and phagocytosis (15). Recent research has found that ELMO3 plays a key role in the metastasis of NSCLC (16, 17). However, the mechanism of EMLO3 in NSCLC remains unclear. In our study, mRNA and protein expression of ELMO3 was increased significantly in NSCLC tissues and cell lines. These findings are consistent with previous lung cancer studies in which expression of ELMO3 was up-regulated in the lung and serum of patients with NSCLC. The expression of ELMO3 was associated with a number of clinical pathological features and can be used as a potential diagnostic and prognostic indicator (13). In addition, studies have reported that the expression of ELMO3 in patients with distant metastases was significantly higher than that in patients without distant metastases in NSCLC patients, and therefore ELMO3 may be a direct driver of cancer metastasis (10, 17). This further suggests that inhibition of ELMO3 can inhibit the metastasis of NSCLC.

Adhesion and transwell experiments demonstrated that ELMO3 siRNA inhibited adhesion and invasion of LLC cells by inhibiting EMT. Activation of EMT signaling in cancer cells is widely considered to contribute to metastasis, relapse or treatment resistance (18). Targeting EMT signaling is considered as new therapeutic strategies for lung cancer (18). The loss of the epithelial marker E-cadherin in lung cancer has been associated with advanced histological grade and metastasis (19). Herein, we found that siELMO3 could inhibit the protein expression of N-cadherin, Snail, and Vimentin, and upregulation of the E-cadherin in LLC cells (Figure 2E). Nonetheless, our current data suggest that siELMO3 could inhibit cell invasion and migration by suppressing EMT in LLC cells.

There is compelling evidence that COX-2 has a potential role in lung cancer therapeutics (20). Our study showed that ELMO3 expression was positively correlated with COX-2 expression in NSCLC tissues (Figure 1D). Previous research found that COX-2 interacted with ELMO1 confirmed by co-immunoprecipitation (co-IP). Since ELMO3 and ELMO1 have similar structures, including the ELMO domain, ras GTPase-binding domain (RBD), ELMO Inhibitor domain (EID), ELMO autoregulatory domain (EAD), the extreme C-terminal proline-rich motifs and PH domain (4, 15, 21), then ELMO3 may regulate NSCLC through similar mechanisms. In addition, COX-2 inhibitors can significantly down-regulate the expression of ELMO3. Therefore, the process of ELMO3 regulation of tumor growth and metastasis may be controlled by COX-2. However, this study is only a preliminary discussion of the relationship between COX-2 and ELMO3, and the specific regulatory mechanisms need to be further studied.

This study has established a new therapeutic target ELMO3 for lung cancer treatment. Unfortunately, ELMO3 does not have clinically safe inhibitors. Our study found that parecoxib can significantly inhibit the expression of ELMO3. Parecoxic inhibited the protein expression of N-cadherin, Snail, and Vimentin, and up-regulate of the expression of E-cadherin in LLC cells. Furthermore, Parecoxib significantly inhibited tumor growth and EMT in LLC-bearing mice (Figures 6A–C, E). Parecoxib is commonly used in clinical peri-operative analgesics and is a COX-2 selective inhibitor with excellent safety. Parecoxib is safe when used for more than 3 days for the management of postoperative pain (22). Therefore, our study suggests that Parecoxib can be used as an inhibitor of ELMO3 and used for the treatment of NSCLC.

In conclusion, we have shown that the expression of ELMO3 was observed in the NSCLC specimens. Parecoxib could inhibit the growth, adhesion, EMT and metastasis of lung cancer cells by downregulating ELMO3. Therefore, Parecoxib may serve as a tool to study ELMO3 inhibition, and might be a potential candidate for the treatment of NSCLC, inhibiting tumor metastasis and preventing tumor recurrence.

## Ethics Statement

All procedures described in this study were performed in accordance to the guidelines of Institutional Animal Ethical Committee of Nanjing Medical University and in accordance with the ethical standards established by Ethics Committee of Zhangjiagang Hospital of Traditional Chinese Medicine affiliated to Nanjing University of Chinese Medicine. The protocol that involving human subjects, was approved by Ethics Committee of Zhangjiagang Hospital of Traditional Chinese Medicine affiliated to Nanjing University of Chinese Medicine. The protocol that involving animal subjects, was approved by Institutional Animal Ethical Committee of Nanjing Medical University.

## Author Contributions

CP carried out the experiments, assisted with data analysis, and helped in manuscript preparation. YZ did the Real Time-PCR experiment and Immunohistochemical detection. QM provided technical assistance in slicing and staining experiments. GD participated in the design of the study and took care of the experimental animals. ZJ collected clinical samples and assisted with manuscript preparation. HB conducted the writing of the manuscript and all the experiments.

### Conflict of Interest Statement

The authors declare that the research was conducted in the absence of any commercial or financial relationships that could be construed as a potential conflict of interest.
